# Cystatin B, cathepsin L and D related to surrogate markers for cardiovascular disease in children

**DOI:** 10.1371/journal.pone.0187494

**Published:** 2017-11-17

**Authors:** Magnus Dencker, Tina Tanha, Magnus K. Karlsson, Per Wollmer, Lars B. Andersen, Ola Thorsson

**Affiliations:** 1 Dept of Translational Medicine, Unit of Medical Imaging and Physiology, Skåne University Hospital, Lund University, Malmö, Sweden; 2 Dept of Clinical Sciences, Clinical and Molecular Osteoporosis Research Unit, Skåne University Hospital, Lund University, Malmö, Sweden; 3 Sogn and Fjordane University College, Sogndal, Norway; 4 Department of Sports Medicine, Norwegian School of Sport Sciences, Oslo, Norway; John Hopkins University School of Medicine, UNITED STATES

## Abstract

**Objective:**

This study investigated potential associations between novel biomarkers for cardiovascular disease and other surrogate markers for health.

**Methods:**

Community sample of 170 (92 boys and 78 girls) children aged 8–11 years. Total fat mass (TBF) and abdominal fat (AFM) were measured by Dual-energy x-ray absorptiometry (DXA). Total body fat was also expressed as percentage of total body mass (BF%), and body fat distribution was calculated as AFM/TBF. Maximal oxygen uptake (VO_2PEAK_), systolic and diastolic blood pressure (SBP and DBP) and pulse pressure (PP) were measured. Echocardiography was performed. Left atrial size (LA) and left ventricular mass (LVM) were measured. A follow-up DXA scan was available in 152 children (84 boys and 68 girls). Frozen serum samples were analyzed for cystatin B, cathepsin L and cathepsin D.

**Results:**

Partial correlations between cystatin B versus lnTBF, lnBF%, lnAFM, AFM/TBF, VO_2PEAK_ and PP were; r = 0.38, 0.36, 0.38, 0.29, -0.25 and 0.25, P = 0.001 or less for all. Weaker predominantly non-significant correlations were found for cathepsin L, whereas cathepsin D was not related to any surrogate markers for health. No significant correlations were found between biomarkers and change in body fat over 2 years.

**Conclusion:**

Findings from this community-based cohort of young children show that surrogate markers for cardiovascular disease such as total fat mass, percent body fat, abdominal fat, body fat distribution, maximal oxygen uptake and pulse pressure were all associated with cystatin B. This was not found for cathepsin L or cathepsin D.

## Introduction

Cathepsin D, cathepsin L and cystatin B, the inhibitor of cathepsin L, belong to a family of proteolytic enzymes, which are supposed to be present only in small amounts in normal arteries, whereas increased presence have been detected within atherosclerotic lesions [1–3]. Although they have all been proposed to be potential biomarkers for cardiovascular disease (CVD), the number of studies in adults is limited. Cathepsin L has been investigated in mainly patient cohorts [4–6], and also cystatin B in a small patient cohort [7]. Recently it was shown that cathepsin D, cathepsin L and cystatin B are associated with increased incidence of CVD in a large population based sample [8]. There are little data in children concerning cathepsin D, cathepsin L and cystatin B. Mutations in the gene which encodes cystatin B have been shown to be responsible for a very rare form of progressive myoclonus epilepsy [9], and cathepsin D levels has been suggested to be a potential marker for pediatric hepatic inflammation [10]. Cathepsin L has been studied in Ewing sarcoma [11] and has been shown to predict kidney dysfunction in children with type 1 diabetes [12]. The purpose of this investigation was, in a community sample of children, to assess possible relationships between cathepsin D, cathepsin L and cystatin B versus known risk factors for CVD such as total body fat, abdominal fat, body fat distribution, aerobic fitness, blood pressure, left ventricular mass and left atrial size and if these biomarkers had any relations with increase in body fat over 2 years.

## Material and methods

### Subjects

The study population was recruited among children at four different schools in Malmö, Sweden. The schools were all situated in socially homogeneous middle-class areas with inhabitants of essentially non-immigrant origin. All 477 children (boys = 259, girls = 218) attending third or fourth grade were invited to participate in the study. Out of these 477 children 248 accepted the invitation (boys = 140, girls = 108), and 172 children gave blood samples (it was optional to do so). Blood samples and a complete data set of other variables were available in 170 children (92 boys and 78 girls) resulting in an inclusion frequency of 36%. A 2-year follow-up DXA scan was available in 152 children (84 boys and 68 girls). Written informed consent was obtained from the parents of all participating children. The research ethics committee at Lund University, Sweden, approved the study (LU 243–01).

### Anthropometric measures

Anthropometric measures were performed at baseline and at the follow-up measurement (mean±SD) 2.0±0.1 years later. Total body height was measured to the nearest 1 cm using a fixed stadiometer (Hultafors AB, Hultafors, Sweden) and total body mass was measured to the nearest 1 kg with a standardized scale (Avery Berkel model HL 120, Avery Weigh-Tronix Inc, Fairmont, MN, USA). The children were dressed in light clothing. Puberty status was assessed by self-evaluation according to Tanner [13].

### Dual-energy x-ray absorptiometry (DXA)

Body composition was measured by DXA total body scan (DPX-L version 1.3z, Lunar, Madison, WI, USA), both at baseline and at the follow-up measurement. This method uses two beams of low-energy X-rays that are collected by the external detector. Soft tissue is resolved by use of mass attenuation coefficients derived from tissue-equivalent standards for fat-free and fat tissue. Pediatric software was used for children with a weight below 30 kg. Daily calibration of the machines was carried out with the Lunar phantom. One research technician performed all measurements and software analyses. Total body fat mass (TBF) and abdominal fat (AFM) were quantified. Percent body fat (BF%) was calculated as percentage of total body mass. Body fat distribution was calculated as AFM/TBF. Studies have shown that DXA provides accurate measurements of fat mass, including regional fat mass [14].

### Measurement of aerobic fitness

Maximal oxygen uptake (VO_2PEAK_) was determined by a maximum exercise test at the baseline. The test was performed on an electrically braked bicycle ergometer (Rodby rhc, model RE 990, Rodby Innovation AB, Karlskoga, Sweden). Expired gas was sampled continuously via a mixing chamber and analyzed for the concentration of O_2_ and CO_2_ (Sensor Medics 2900, SensorMedics Inc, Yorba Linda, CA, USA). Measurements were obtained every 20 s during 2 min at rest and during exercise with progressively increasing workload to volitional exhaustion. All children, regardless of gender, fitness, height and body mass, used the same protocol with an initial workload of 30 Watt (W) and an increase of 15 W per minute. Maximum heart rate (max HR) and maximum respiratory exchange ratio (RER) were recorded. VO_2PEAK_ was determined as the highest value during the last minute of exercise and scaled to body mass (ml/min/kg). The exercise test was considered acceptable if it met one of the following criteria: RER ≥ 1.0, max HR >90% of predicted value or signs of intense effort (e.g. hyperpnoea, facial flushing or inability to keep adequate revolutions/min (53–64)) [15].

### Blood pressure

A Dinamap paediatric vital signs monitor (model XL, Critikron Inc., Tampa, FL, USA) was used to measure systolic blood pressure (SBP), and diastolic blood pressure (DBP) in the seated position after 15 minutes of rest [16]. The mean of three measurements was used in all analyses. Pulse pressure (PP) was calculated as SBP-DBP.

### Echocardiography

Echocardiographic examination was performed with subjects in the left lateral recumbent position using Sonos 2500 (Philips Inc, Eindhoven, the Netherlands) or Aspen (Acuson Inc, Mountain View, CA, USA) equipment. Studies were performed with 2-dimensional guided M-mode echocardiography obtained in the parasternal short and long-axis views, in accordance with the American Society of Echocardiography (ASE) recommendations [17]. The following variables were measured: End-diastolic left ventricular diameter (LVDD), left atrial end-systolic diameter (LA), end-diastolic inter-ventricular septum thickness (IVS), and end-diastolic posterior wall thickness (PW). Left ventricular mass (LVM) was calculated using the ASE convention: LVM = 0.83 x [(LVDD + IVS + PW)3—LVDD^3^] + 0.6 (measurements in cm) [17]. Both LVM and LA were indexed for height [18–20].

### Blood samples

Non-fasting venous serum sample were drawn from 172 children and stored at –70°C until analysis. The samples were collected in conjunction with the DXA measurement. Cystatin B, cathepsin D and cathepsin L were analyzed by the Proximity Extension Assay technique using the Proseek Multiplex CVD 96x96 reagents kit (Olink Bioscience, Uppsala, Sweden) at the Clinical Biomarkers Facility, Science for Life Laboratory, Uppsala, as previously described [21,22]. The variance for intra-assay variation and inter-assay variation cystatin B, cathepsin D and cathepsin L were 8% and 13%, 8% and 16%, 7% and 14%, respectively. Data are presented as arbitrary units (AU). Values can be transformed to actual concentrations using transforms on the Olink Bioscience website. There is, however, an approximation in this transformation. Two samples were excluded due to technical problems at the analysis.

### Statistical analyses

All analyses were made in Statistica 12 (StatSoft Inc, Tulsa, OK, USA). The descriptive data are presented as median and interquartile range. Distribution of body fat measurements and abdominal fat were skewed and therefore normalized by natural logarithm (ln). Differences in anthropometric data between boys and girls were tested using Student’s t-test for continuous variables and Mann–Whitney U test was used for ordinal data. Unadjusted relations between cystatin B and main outcome (ln total body fat, ln abdominal fat, body fat distribution, and fitness) was assessed with scatterplots and Pearson correlation analysis. Partial correlations for all children, with adjustment for sex, age, and school location between the biomarkers and lnTBF, lnAFM, AFM/TBF, VO_2PEAK_, SBP, DBP, PP, LVM and LA were assessed by regression analysis. Additional regression analysis were also made between the biomarkers and physiological measurements (fitness, SBP, DBP, PP, LVM, LA) with adjustments for ln Total body fat. Regression analysis was also used to assess partial correlations between biomarkers and increase in TBF, AFM, BF% or change AFM/TBF. In these analyses adjustment for sex, age, school location and change in Tanner stage were made. Tanner stage score was included as a factor variable. In the regression analyses we only consider p-values 0.001 or less as significant to counteract potential problems with multiple testing.

## Results

**Fig 1:**
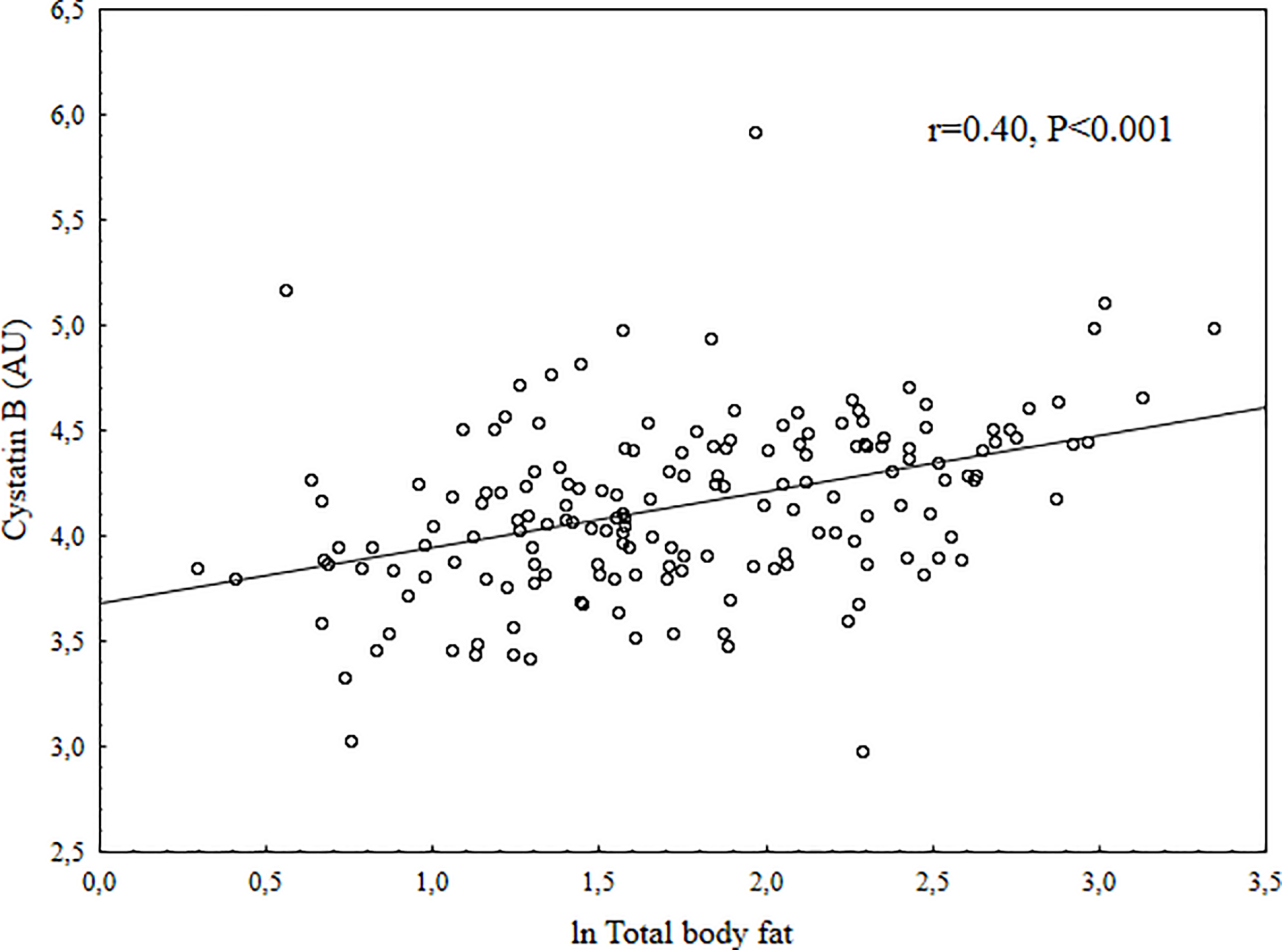
Scatter plot between Cystatin B in arbitrary units (AU) and ln total body fat.

**Fig 2:**
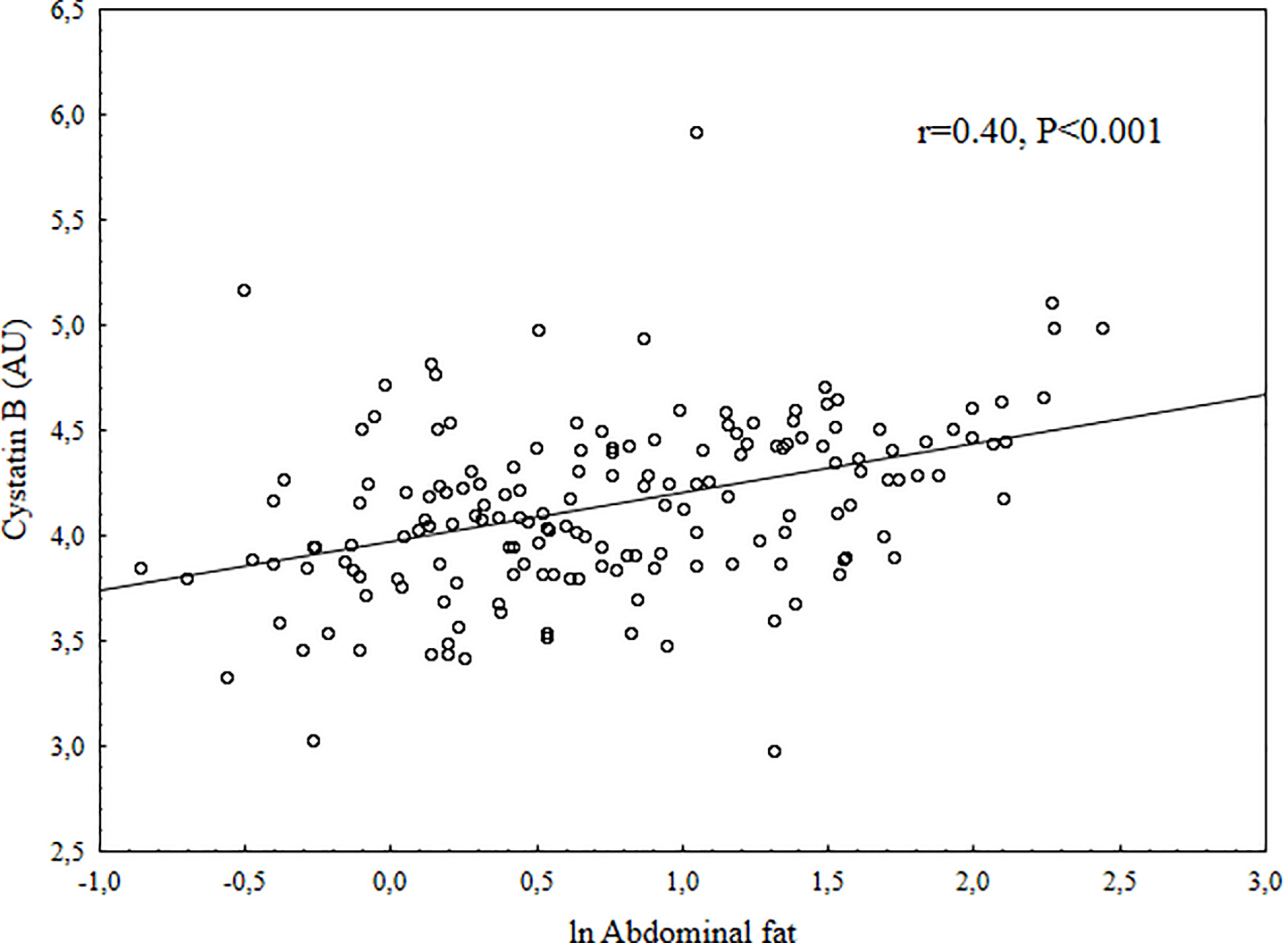
Scatter plot between Cystatin B in arbitrary units (AU) and ln abdominal fat.

**Fig 3:**
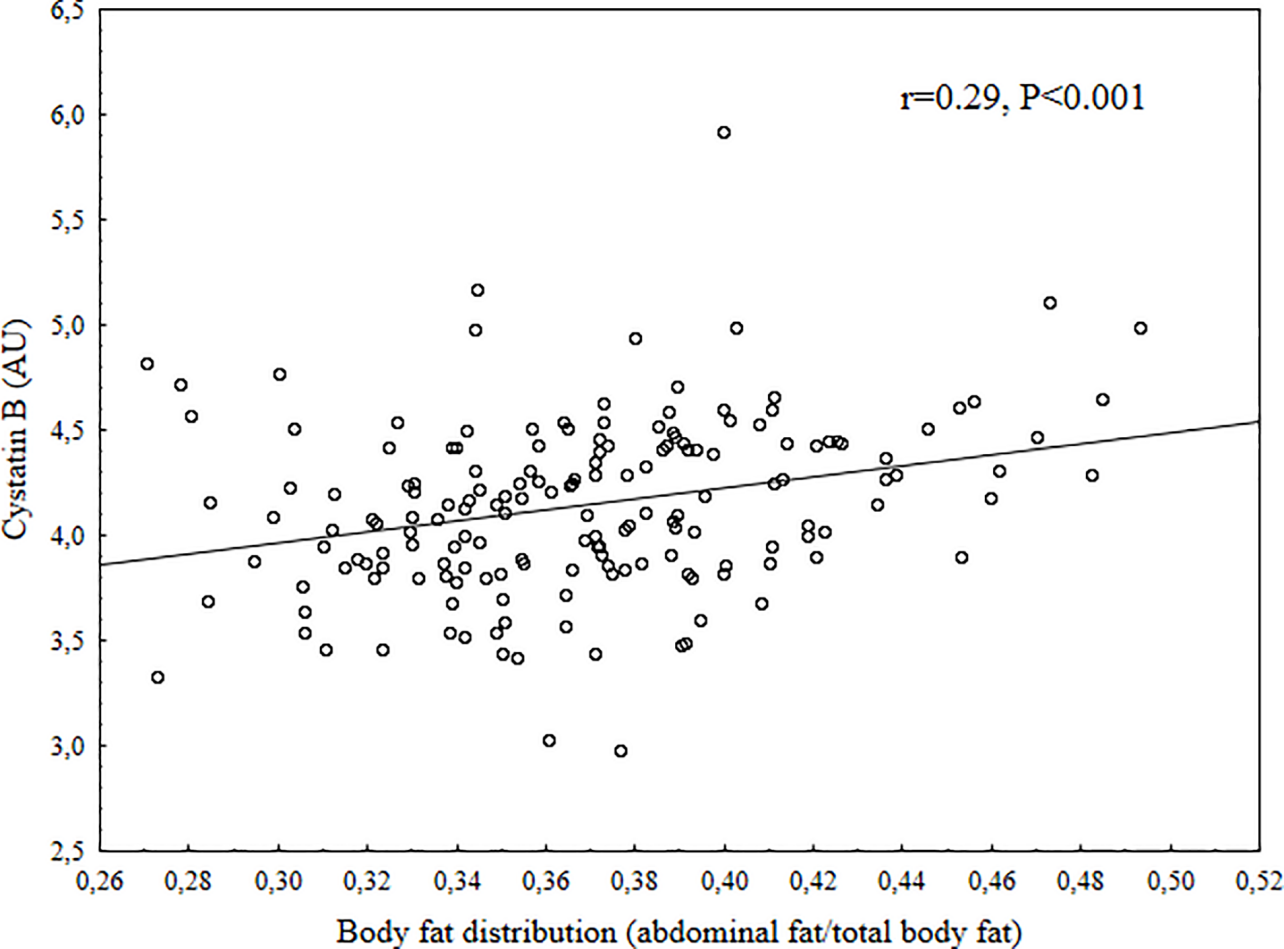
Scatter plot between Cystatin B in arbitrary units (AU) and body fat distribution (abdominal fat/total body fat).

**Fig 4:**
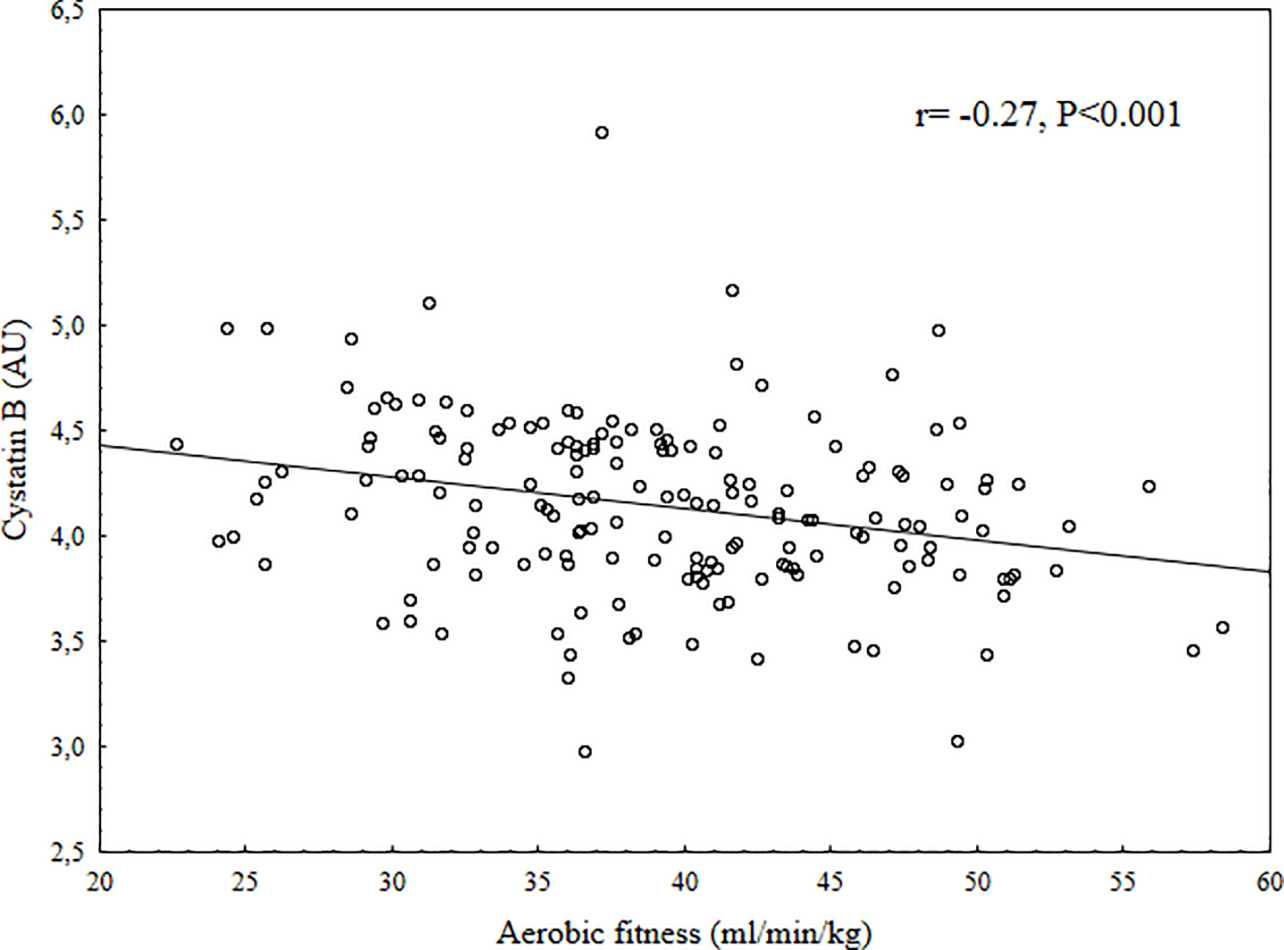
Scatter plot between Cystatin B in arbitrary units (AU) and fitness (ml/min/kg).

Children that did not consent to give a blood sample were younger than those who gave consent (9.5 vs. 9.9 years, P = 0.003), no other differences were found in anthropometric, fitness or DXA data between those children who were part of the final study group at baseline and those who were not (S1 Table). The children who did not participate in the follow-up were younger at baseline than those who did (9.6 vs. 10.0 years, P = 0.004). There were no differences in anthropometrics, fitness or DXA data between those who participated in the follow-up and those who did not (S2 Table). At baseline three girls were Tanner stage 2, all other children Tanner stage 1. The distribution of Tanner score at follow-up was; (N for stage 1 = 18, stage 2 = 63, stage 3 = 49, stage 4 = 22). At baseline, there was no significant differences between boys and girls except girls had higher TBF, BF% and AFM and boys had higher fitness and more LVM. At follow-up there were no differences in increase of body fat measurement except that boys had a higher increase in BF%. Girls had higher Tanner score at follow-up. Summary of baseline and follow-up data are displayed in Table 1. Unadjusted correlations between cystatin B and main outcome indicated moderate correlations (ln total body fat (r = 0.40), ln abdominal fat (r = 0.40), body fat distribution (r = 0.29), and fitness (r = -0.27), all P<0.001) (Figs 1–4). Also, multiple regression analysis, with adjustments, indicated moderate relations between cystatin B versus ln TBF, ln BF%, ln AFM, AFM/TBF, VO_2PEAK_ and PP (Table 2). Cathepsin L had a trend for similar relations with investigated measurements but the relations were overall weaker and for most parts not significant, the only exception was AFM/TBF. In contrast, cathepsin D had no relationships with all the other risk factors for CVD. When additional regression analysis were made and partial correlations between the biomarkers and physiological measurements (fitness, SBP, DBP, PP, LVM, LA) were calculated with adjustments also for ln Total body fat, all significant relationships disappeared (Table 3). No significant correlations were found between cystatin B, cathepsin L or cathepsin D and change in body fat over 2 years. Summary of the relationships between biomarkers and follow-up data in Table 4.

**Table 1:** Display of age, anthropometric, fitness and Tanner statistics median (interquartile range) for baseline and follow-up measurements. Total body fat (TBF), abdominal fat (AFM), maximum heart rate (Max HR), respiratory exchange ratio (RER), systolic blood pressure (SBP), diastolic blood pressure (DBP), and biomarkers.

Variable	Boys (n = 92)	Girls (n = 78)	P-value
**Baseline**			
Age (yrs)	10.2 (0.8)	9.7 (1.0)	0.03
Height (cm)	141 (9)	140 (10)	0.50
Body mass (kg)	34.0 (8.0)	34.0 (9.0)	0.57
BMI (kg/m^2^)	16.9 (3.1)	17.1 (3.5)	0.90
Total body fat (kg)	4.6 (5.1)	6.7 (5.3)	<0.001
Percent body fat (%)	13.7 (11.1)	21.1 (11.2)	<0.001
Abdominal fat (kg)	1.6 (2.1)	2.5 (2.4)	<0.001
Fat distribution (AFM/TBF)	0.36 (0.05)	0.4 (0.1)	0.09
Fitness (ml/min/kg)	42 (11)	36 (8)	<0.001
Max HR (beats/min)	190 (21)	188 (22)	0.03
RER	1.0 (0.1)	1.0 (0.1)	0.64
SBP (mmHg)	104 (12)	104 (12)	0.75
DBP (mmHg)	60 (8)	60 (8)	0.48
Pulse pressure (mmHg)	45 (10)	43 (6)	0.37
Left ventricular mass (g/m)	51.7 (17.1)	48.1 (17.3)	0.002
Left atrial diameter (mm/m)	19.9 (2.7)	19.4 (3.1)	0.22
Cystatin B (AU)	4.0 (0.5)	4.2 (0.5)	0.01
Cathepsin L (AU)	4.8 (0.3)	4.9 (0.2)	0.49
Cathepsin D (AU)	6.9 (0.4)	6.9 (0.4)	0.87
Tanner stage score	1.0 (0.0)	1.0 (0.0)	0.67
**Follow-up**	**Boys (n = 84)**	**Girls (n = 68)**	
Age (yr)	12.1 (0.8)	11.7 (1.1)	0.16
Height (cm)	152 (92)	154 (12.3)	0.31
Body Mass (kg)	43.2 (12.3)	42.2 (16.5)	0.62
BMI (kg/m^2^)	18.4 (3.3)	17.9 (4.5)	0.97
Increase in TBF (kg)	1.5 (3.5)	2.0 (3.0)	0.95
Increase in AFM (kg)	0.6 (1.5)	0.9 (1.7)	0.63
Increase in percent body fat (%)	1.0 (5.6)	0.2 (4.3)	0.03
Change in fat distribution	0.0 (0.0)	0.0 (0.0)	0.32
Tanner stage score	2.0 (1.0)	3.0 (1.0)	0.007

**Table 2:** Partial correlations (R) between biomarkers versus different baseline measurements. Adjustment made for age (not for partial correlations with age), sex, and school location.

Variable	Cystatin B	Cathepsin L	Cathepsin D
	R b (se_b_) p-value	R b (se_b_) p-value	R b (se_b_) p-value
Age (years)	-0.04 -0.030 (0.050) 0.56	0.03 0.077 (0.002) 0.68	-0.15 -0.068 (0.034) 0.05
Height (m)	0.13 0.008 (0.005) 0.09	0.02 0.004 (0.001) 0.78	0.04 0.002 (0.003) 0.60
Body mass (kg)	0.38 0.022 (0.004) <0.001	0.15 0.001 (0.010) 0.05	0.11 0.005 (0.003) 0.13
BMI (kg/m^2^)	0.41 0.061 (0.011) <0.001	0.18 0.013 (0.005) 0.02	0.11 0.011 (0.008) 0.14
ln Total body fat (TBF)	0.38 0.249 (0.047) <0.001	0.18 0.060 (0.025) 0.02	0.11 0.050 (0.034) 0.14
ln Percent body fat	0.36 0.322 (0.064) <0.001	0.19 0.083 (0.033) 0.01	0.11 0.067 (0.046) 0.15
ln Abdominal fat (AFM)	0.38 0.219 (0.041) <0.001	0.21 0.059 (0.022) 0.008	0.12 0.047 (0.030) 0.12
Fat distribution (AFM/TBF)	0.29 2.571 (0.666) <0.001	0.25 1.123 (0.337) 0.001	0.12 0.719 (0.464) 0.12
Fitness (ml/min/kg)	-0.25 -0.015 (0.005) 0.001	-0.05 -0.001 (0.002) 0.01	-0.06 -0.003 (0.003) 0.43
Systolic blood pressure (mmHg)	0.19 0.009 (0.004) 0.01	0.10 0.002 (0.002) 0.19	0.06 -0.054 (0.018) 0.44
Diastolic blood pressure (mmHg)	-0.04 -0.003 (0.006) 0.56	-0.09 -0.003 (0.003) 0.27	0.01 0.001 (0.004) 0.85
Pulse pressure (mmHg)	0.25 0.013 (0.004) 0.001	0.18 0.005 (0.002) 0.02	0.05 0.002 (0.003) 0.48
Left ventricular mass (g/m)	0.17 0.006 (0.003) 0.03	0.15 0.003 (0.001) 0.06	0.07 0.002 (0.002) 0.39
Left atrial diameter (mm/m)	0.10 0.018 (0.014) 0.20	0.18 0.016 (0.007) 0.02	0.00 0.000 (0.009) 0.96

**Table 3:** Partial correlations (R) between biomarkers versus different baseline measurements. Adjustment made for age, sex, school location and ln total body fat.

Variable	Cystatin B	Cathepsin L	Cathepsin D
	R b (se_b_) p-value	R b (se_b_) p-value	R b (se_b_) p-value
Fitness (ml/min/kg)	-0.02 -0.001 (0.004) 0.82	0.08 0.003 (0.003) 0.30	0.01 0.000 (0.004) 0.90
Systolic blood pressure (mmHg)	0.04 0.002 (0.004) 0.54	0.03 0.001 (0.002) 0.69	0.02 0.001 (0.003) 0.85
Diastolic blood pressure (mmHg)	-0.12 -0.008 (0.005) 0.13	-0.12 -0.004 (0.003) 0.12	0.00 0.000 (0.004) 0.96
Pulse pressure (mmHg)	0.14 0.007 (0.004) 0.07	0.12 0.003 (0.002) 0.11	0.02 0.001 (0.003) 0.80
Left ventricular mass (g/m)	0.05 0.002 (0.003) 0.54	0.09 0.002 (0.002) 0.23	0.03 0.001 (0.002) 0.69
Left atrial diameter (mm/m)	0.03 0.006 (0.013) 0.65	0.15 0.014 (0.007) 0.048	-0.02 -0.002 (0.010) 0.83

**Table 4:** Partial correlations (R) between biomarkers versus change in body fat measurements at follow-up. Adjustment made for age, sex, school location, and change in Tanner stage score.

Variable	Cystatin B	Cathepsin L	Cathepsin D
	R b (se_b_) p-value	R b (se_b_) p-value	R b (se_b_) p-value
Increase in TBF (kg)	0.16 0.026 (0.013) 0.04	0.17 0.014 (0.006) 0.03	0.15 0.016 (0.009) 0.08
Increase in AFM (kg)	0.15 0.045 (0.024) 0.06	0.10 0.015 (0.012) 0.23	0.14 0.028 (0.016) 0.09
Increase in percent body fat	0.00 0.000 (0.008) 0.96	0.08 0.004 (0.004) 0.32	0.11 0.008 (0.006) 0.32
Change in fat distribution	0.06 0.232 (0.320) 0.47	0.00 0.000 (0.021) 0.96	0.02 0.045 (0.217) 0.84

## Discussion

The present study is, to our knowledge, the first to establish associations between cystatin B levels and total body fat, abdominal fat, body fat distribution, fitness and pulse pressure in unselected young children. Cathepsin L had similar relations but overall weaker and non-significant relations. Cathepsin D had no relationships with any other risk factors for CVD. The relationships found between the biomarkers and physiological measurements such as fitness and pulse pressure seems to be driven by body fat. When we calculated partial correlations with adjustments also for total body fat, all significant relationships disappeared.

There are, to our knowledge, no data in children on potential relationships between these novel biomarkers for CVD versus other traditional risk factors for CVD. In adults, cathepsin D, cathepsin L and cystatin B have been investigated as biomarkers for CVD [4–8]. One hospital-based study found a rather strong correlation of serum cathepsin L levels and the degree of stenosis in the left anterior descending coronary artery patients with coronary artery stenosis and that subjects with stenosis had higher cathepsin L levels than subjects without [4]. Another study found higher cathepsin L in individuals who had acute and previous myocardial infarction or stable and unstable angina pectoris compared to controls, and that cathepsin L correlated moderately with the number of stenotic lesions [5]. In addition, plasma cathepsin L was found to be higher in patients with abdominal aortic aneurysm [6]. Recently cystatin B, cathepsin L and cathepsin D was established as novel risk factors for CVD in the general population [8]. Our findings of a weak to moderately strong association between cystatin B and several investigated risk factors for CVD suggest that the risk factor exposure starts early. The findings for cathepsin L were less pronounced and for most parts not significant. The finding for cathepsin D was completely negative. There is no definitive explanation to these discrepancies. Cathepsin D, cathepsin L and cystatin B are all supposed to be present only in small amounts in normal arteries and increase when atherosclerotic lesions have been formed [1–3]. Data from autopsy studies of the Bogalusa Heart Study, in children and adolescent aged 2–15 years, indicated a prevalence of fibrous-plaque lesions in the aorta of about 20% and slightly less than 10% in the coronary arteries [23]. But these fibrous-plaque lesions should be considered precursors and not a fully-fledged atherosclerotic lesion and it is possible that those precursors produce very little Cathepsin D, which may explain that no relations with traditional risk factors were found. A slightly different case could be suggested for cathepsin L, where the relations could perhaps be affected by the fact that elevation of cathepsin L levels is counteracted by its inhibitor cystatin B. There might be a completely different explanation for our findings concerning cystatin B. Cystatin B is perhaps not just produced in arteries in general and in atherosclerotic lesions in particular, but also perhaps produced by fat cells. This is purely speculative, as we have not been able to find any clinical or experimental data to support this theory. However, Goncalves and co-workers also found a significant correlation between cystatin B, cathepsin L and cathepsin D versus BMI in their study [8]. These correlations were, however, weaker (r = 0.12–0.22) than the one we found.

Major strengths of the present investigation are the urban community-based sample, the multitude of measurements, and objective measurement of body fat by DXA. Although DXA measurement of fat mass is a much more accurate estimate of adiposity than anthropometric measurements, DXA is only capable of measuring total abdominal fat, and is not able to differentiate between intra-visceral and extra-visceral fat.

The inclusion frequency in this study of 52% might be considered somewhat low. A complete data set was available in no more than 36% (170 out of 477 invited children). A separate study of anthropometric data, from school nurses, for all children that received an invitation to participate in the study (n = 477) showed no significant differences in height, body mass or BMI between the children that chose to participate (n = 248) and those who did not [24]. Moreover, there were no major differences (except an age difference) in baseline measurements between those who accepted blood sampling and those who participated in the follow-up versus those who did not. We, however, acknowledge that the dropout is a not fully quantifiable limitation of the study. There is also the problem with potential confounders. In the regression analysis we adjusted for sex, age, school location and change in Tanner stage at follow-up as these factors varied. Statistical adjustment may not necessarily compensate completely for biological differences. The fact that we had non-fasting blood samples has not biased the analysis as it has been shown that cystatin B, cathepsin D or cathepsin L levels are not altered by food intake [25].

## Conclusions

The present community-based study of young children shows that increased body fat and abdominal fat, more abdominal body fat distribution, low fitness, and higher pulse pressure were associated with increased levels of cystatin B. Change in body fat over 2 years was not significantly associated with cystatin B, cathepsin D or cathepsin L levels. Cathepsin L had similar relations but overall weaker, and for most part non-significant, relations than cystatin B, whereas cathepsin D had no relationships with the other risk factors for CVD.

## Supporting information

S1 Table Display of sex, age, anthropometric, fitness and Tanner statistics median (interquartile range) for baseline measurements between participants who had blood samples and those who had not. (DOCX)

S2 Table Display of sex, age, anthropometric, fitness and Tanner statistics median (interquartile range) for baseline measurements between participants who participated in the follow-up and those who did not. (DOCX)
