# Supplementary material for: Cystatin B, cathepsin L and D related to surrogate markers for cardiovascular disease in children
Source: PLoS One. 2017 Nov 17;12(11):e0187494. doi: 10.1371/journal.pone.0187494 (PMC5693439; doi:10.1371/journal.pone.0187494)
Supplement: S1 Table — (DOCX) [file pone.0187494.s001.docx]

**S1 Table.** Display of sex, age, anthropometric, fitness and Tanner statistics median (interquartile range) for baseline measurements between participants who had blood samples and those who had not. Total body fat (TBF), abdominal fat (AFM), maximum heart rate (Max HR), respiratory exchange ratio (RER), systolic blood pressure (SBP), and diastolic blood pressure (DBP).

| **Variable** | **Participants (n=170)** | **Non-participants (n=78)** | **P-value** |
| --- | --- | --- | --- |
| Boys/Girls | 92/78 | 48/30 | 0.35 |
| Age (yrs) | 9.9 (0.9) | 9.5 (1.0) | 0.003 |
| Height (cm) | 141 (10) | 141 (11) | 0.51 |
| Body mass (kg) | 34.0 (10.0) | 32.0 (10.0) | 0.84 |
| BMI (kg/m^2^) | 17.0 (3.2) | 16.5 (3.0) | 0.91 |
| Total body fat (kg) | 5.5 (6.0) | 4.9 (4.6) | 0.60 |
| Percent body fat (%) | 17.1 (13.1) | 15.6 (12.6) | 0.87 |
| Abdominal fat (kg) | 1.9 (2.5) | 1.8 (2.1) | 0.47 |
| Fat distribution (AFM/TBF) | 0.37 (0.05) | 0.36 (0.07) | 0.84 |
| Fitness (ml/min/kg) | 39 (10) | 39 (10) | 0.55 |
| Max HR (beats/min) | 187 (19) | 186 (28) | 0.99 |
| RER | 1.0 (0.1) | 1.0 (0.1) | 0.42 |
| SBP (mmHg) | 104 (12) | 105 (13) | 0.18 |
| DBP (mmHg) | 60 (8) | 60 (8) | 0.86 |
| Pulse pressure (mmHg) | 44 (8) | 45 (10) | 0.10 |
| Left ventricular mass (g/m) | 50.4 (16.4) | 49.0 (16.2) | 0.19 |
| Left atrial diameter (mm/m) | 19.8 (3.0) | 20.0 (3.5) | 0.59 |
| Tanner stage score | 1.0 (0.0) | 1.0 (0.0) | 0.92 |
