# Supplementary material for: Cystatin B, cathepsin L and D related to surrogate markers for cardiovascular disease in children
Source: PLoS One. 2017 Nov 17;12(11):e0187494. doi: 10.1371/journal.pone.0187494 (PMC5693439; doi:10.1371/journal.pone.0187494)
Supplement: S2 Table — (DOCX) [file pone.0187494.s002.docx]

**S2 Table.** Display of sex, age, anthropometric, fitness and Tanner statistics median (interquartile range) for baseline measurements between participants who participated in the follow-up and those who did not. Total body fat (TBF), abdominal fat (AFM), maximum heart rate (Max HR), respiratory exchange ratio (RER), systolic blood pressure (SBP), and diastolic blood pressure (DBP).

| **Variable** | **Participants (n=152)** | **Non-participants (n=96)** | **P-value** |
| --- | --- | --- | --- |
| Boys/Girls | 84/68 | 56/40 | 0.64 |
| Age (yrs) | 10.0 (0.9) | 9.6 (1.0) | 0.004 |
| Height (cm) | 141 (10) | 141 (10) | 0.97 |
| Body mass (kg) | 34.0 (10.0) | 33.0 (8.5) | 0.70 |
| BMI (kg/m^2^) | 17.0 (3.3) | 16.6 (3.0) | 0.88 |
| Total body fat (kg) | 5.5 (6.1) | 4.9 (5.3) | 0.35 |
| Percent body fat (%) | 17.0 (13.6) | 15.8 (12.2) | 0.63 |
| Abdominal fat (kg) | 1.9 (2.5) | 1.8 (2.2) | 0.29 |
| Fat distribution (AFM/TBF) | 0.37 (0.05) | 0.37 (0.07) | 0.67 |
| Fitness (ml/min/kg) | 39 (10) | 39 (10) | 0.52 |
| Max HR (beats/min) | 188 (18) | 189 (27) | 0.45 |
| RER | 1.0 (0.1) | 1.0 (0.1) | 0.30 |
| SBP (mmHg) | 104 (12) | 105 (12) | 0.10 |
| DBP (mmHg) | 60 (8) | 60 (8) | 0.83 |
| Pulse pressure (mmHg) | 44 (8) | 45 (10) | 0.05 |
| Left ventricular mass (g/m) | 50.5 (16.6) | 49.0 (16.1) | 0.08 |
| Left atrial diameter (mm/m) | 19.8 (2.9) | 19.9 (3.5) | 0.80 |
| Tanner stage score | 1.0 (0.0) | 1.0 (0.0) | 0.33 |

**Supplemental table 3.** Partial correlations (R) between biomarkers versus different baseline measurements. Adjustment made for age, sex, school location and ln Total body fat.

| **Variable** | **Cystatin B**  **R**  **b (se_b_)**  **p-value** | **Cathepsin L**  **R**  **b (se_b_)**  **p-value** | **Cathepsin D**  **R**  **b (se_b_)**  **p-value** |
| --- | --- | --- | --- |
| Fitness (ml/min/kg) | -0.02  -0.001 (0.004)  0.82 | 0.08  0.003 (0.003)  0.30 | 0.01  0.000 (0.004)  0.90 |
| Systolic blood pressure (mmHg) | 0.04  0.002 (0.004)  0.54 | 0.03  0.001 (0.002)  0.69 | 0.02  0.001 (0.003)  0.85 |
| Diastolic blood pressure (mmHg) | -0.12  -0.008 (0.005)  0.13 | -0.12  -0.004 (0.003)  0.12 | 0.00  0.000 (0.004)  0.96 |
| Pulse pressure (mmHg) | 0.14  0.007 (0.004)  0.07 | 0.12  0.003 (0.002)  0.11 | 0.02  0.001 (0.003)  0.80 |
| Left ventricular mass (g/m) | 0.05  0.002 (0.003)  0.54 | 0.09  0.002 (0.002)  0.23 | 0.03  0.001 (0.002)  0.69 |
| Left atrial diameter (mm/m) | 0.03  0.006 (0.013)  0.65 | 0.15  0.014 (0.007)  0.048 | -0.02  -0.002 (0.010)  0.83 |
